# Measurement of purpose in life among Arabic-speaking adolescents: psychometric properties of an Arabic-language version of the Claremont purpose scale (CPS)

**DOI:** 10.3389/fpsyg.2025.1583111

**Published:** 2025-06-18

**Authors:** Amir Noureddine, Souheil Hallit, Diana Malaeb, Fouad Sakr, Mariam Dabbous, Tylor Cosgrove, Sahar Obeid, Feten Fekih-Romdhane, Ahmed A. Moustafa

**Affiliations:** ^1^School of Medicine and Medical Sciences, Holy Spirit University of Kaslik, Jounieh, Lebanon; ^2^Psychology Department, College of Humanities, Effat University, Jeddah, Saudi Arabia; ^3^Applied Science Research Center, Applied Science Private University, Amman, Jordan; ^4^College of Pharmacy, Gulf Medical University, Ajman, United Arab Emirates; ^5^School of Pharmacy, Lebanese International University, Beirut, Lebanon; ^6^Faculty of Society and Design, School of Psychology, Bond University, Gold Coast, QLD, Australia; ^7^Social and Education Sciences Department, School of Arts and Sciences, Lebanese American University, Jbeil, Lebanon; ^8^The Tunisian Center of Early Intervention in Psychosis, Department of Psychiatry “Ibn Omrane”, Razi Hospital, Manouba, Tunisia; ^9^Faculty of Medicine of Tunis, Tunis El Manar University, Tunis, Tunisia; ^10^The Faculty of Health Sciences, Department of Human Anatomy and Physiology, University of Johannesburg, Soweto, Johannesburg, South Africa

**Keywords:** purpose in life, Claremont purpose scale, validation, psychometric properties, adolescents, Arabic

## Abstract

**Background:**

Purpose in life is considered a protective factor related to one's ability to thrive, supporting positive development and overall psychological wellbeing of the adolescent. The main goal of our study is to adapt and validate the Claremont purpose scale (CPS) into the Arabic language, expanding its applicability to Arabic-speaking contexts.

**Methods:**

Data for this cross-sectional study was collected via a Google Form link during November 2023. Lebanese adolescents aged 14 to 18 years (*N* = 793, 62.4% females, mean age of 16.08 ± 1.74 years) were recruited using an online questionnaire and a snowball sampling technique.

**Results:**

The fit of the three-factor structure of CPS scores was satisfactory as indicated by the Confirmatory Factor Analysis. Internal reliability was excellent (α = 0.86; ω = 0.87). Measurement invariance across sex groups was established at the metric, configural, and scalar levels. There was no significant difference between male and female respondents in terms of CPS scores. Finally, CPS total scores were significantly correlated with greater depression-happiness (*r* = 0.43; *p* < 0.001), higher wellbeing (*r* = 0.51; *p* < 0.001), and lower irritability (*r* = –.66; *p* < 0.001), attesting to concurrent validity.

**Conclusion:**

Our results suggest that the CPS in its Arabic version is a valid and reliable measure allowing to detect the sense of purpose in Arabic-speaking adolescents, at least from Lebanon. The new validated scale has potential for future practitioners and researchers who would ought to work on ways of improving the psychological functioning of adolescents drawn from an Arab culture.

## Introduction

A sense of purpose in life is identified and unfolded developmentally. Typically, adolescents do not possess the necessary skills to deeply examine and critically reason about their life's purpose (Bronk et al., 2018). At this stage, identity exploration is widely acknowledged (Wood et al., 2018), and the pursuit of meaning is pivotal to identity development in emerging adults (Pfund et al., 2020). For these reasons, an extensive body of empirical research has been devoted to the understanding of the concept and the development of purpose (Ryff, 1989; George and Park, 2016). Purpose can be defined as a long-term aspiration that reaches beyond the limit of simple personal goals to encompass an intention that is both personally relevant and of impact to the wider world (Damon et al., 2003).

Purpose in life (PIL) includes fundamentally three elements (Damon et al., 2003); the first component is general Meaning in Life, which is the overall perception an individual has regarding what is important and renders life worthwhile. This component of meaning gauges the existential core of purpose (Mcknight and Kashdan, 2009; Park, 2010). It forms the basic level over which the other dimensions of purpose develop, providing an existential substrate for a purposeful life. The second component is Goal-directedness, which highlights the drive and purpose behind working toward long-term important objectives. This aspect captures an individual's dedication to pursuing goals that bring a deeper meaning to their life. Unlike short-term everyday goals that may have different impacts, goal-directedness centers on persistently pursuing objectives that resonate with one's fundamental beliefs and ultimate life aims. The third component is Self-Transcendence or Beyond-the-Self Purpose, which highlights the purposive perspective of being altruistic and outward-looking. The term refers to those aspirations which comprise efforts directed toward making a difference to the larger world; in other words, something more than oneself (Schulenberg et al., 2008). Purpose is a motivational construct, deeply related to a few of the principles of humanistic psychology, and includes contributions beyond personal gain. Although self-transcendence historically had fewer studies than general meaning in life and goal-directedness, it represents a key aspect of a fully actualized purpose and orientation that undergoes a marked shift from individualistic pursuits to a more inclusive and altruistically oriented toward a bigger aim (Glanzer et al., 2018). Recent studies has reported that it may result in a higher personal wellbeing and the cultivation of a positive character compared to the pursuit limited only to self-interest (Bronk and Finch, 2010; Hill et al., 2010; Mariano and Vaillant, 2012; Johnson et al., 2018).

The role of purpose is crucial for the wellbeing of adolescents, providing a foundation for preventive health strategies aimed at improving emotional and physical wellness by enhancing knowledge, attitudes, and behaviors (Romano and Hage, 2000). Purpose is regarded as a protective factor related to one's ability to thrive, supporting positive young people development and wellbeing (Larson et al., 2017). Having a sense of PIL is a predictor of resilience and life satisfaction (Sharma and Yukhymenko-Lescroart, 2022), and is associated with physical wellness, such as sleep enhancement, longevity and better cardiovascular health (Hill and Turiano, 2014; Turner et al., 2017). Moreover, research suggests that a strong feeling of purpose serves as a protective factor for happiness and resilience, while also being linked to lower levels of depression and higher life contentment (Veazey et al., 2023). Furthermore, adolescents who have a defined purpose tend to be less irritable and exhibit good emotional regulation, leading to reduced overall psychopathology (Bronk et al., 2018).

In the study of PIL, the instruments predominately used, such as in the Prosocial Youth Purpose Scale and the Revised Youth Purpose Survey, have often missed or struggled to accurately capture the fundamental dimension of beyond-the-self, or opting instead for lengthy and expensive interviews. Furthermore, the mentioned questionnaires require further assessment of their reliability and validity. The Claremont Purpose Scale (CPS) serves as a reliable instrument that covers all the facets of PIL. The CPS consists of 12 items that are structured around three constructs—goal directedness, beyond-the-self orientation and personal meaning (Bronk et al., 2018). This instrument has demonstrated excellent internal consistency, with Cronbach values ranging from 0.916 to 0.935, as well as strong convergent and discriminant validity with measures of general perceived meaning in life, and discriminant validity with measures of depression showing that the scale distinguishes the measurement of purpose from depression (Bronk et al., 2018). Scoring high on the CPS indicates meeting all-purpose criteria where medium scores signal partial fulfillment and low scores suggest minimal fulfillment. The CPS was adapted and validated in other countries and languages, and showed an efficient method for measuring purpose with strong psychometric properties demonstrated in Western studies (Bronk et al., 2018; Veazey et al., 2023) and Asia (Yuliawati, 2022; Wang et al., 2023). The CPS also demonstrated measurement invariance across both college year and undergraduate school indicating that the CPS can be reliably used to compare PIL among college students across different years of study and across different schools (Anghel et al., 2021). Research has consistently confirmed the CPS's three-factor structure, including personal meaningfulness, goal-directedness, and beyond-the-self, across diverse populations (Bronk et al., 2018; Veazey et al., 2023; Wang et al., 2023). For example, Wu et al. (2024) assessed the CPS with Chinese adolescents and found solid support for the three-factor solution, reporting high internal consistency and excellent model fit (CFI = 0.98, RMSEA = 0.047, SRMR = 0.029). The factor loadings for the three components ranged from 0.65 to 0.94, in line with the original conceptual framework. Notably, cultural variations were identified, especially in the beyond-the-self aspect, with Chinese adolescents scoring higher than their American peers, likely due to the influence of collectivist cultural values (Wu et al., 2024). Additionally, the study uncovered partial measurement invariance across age groups and socio-economic backgrounds, with early adolescents and those from rural areas scoring significantly higher on certain items, such as item 11 (“How important is it for you to make the world a better place in some way?”), compared to middle adolescents and those from urban settings. These outcomes highlight the necessity for culturally sensitive adaptations of the scale, especially in non-Western contexts (Wu et al., 2024). Veazey et al. (2023) confirmed the three-factor structure through CFA, reporting factor loadings between 0.65 and 0.94. These results further support the internal consistency of each subscale. However, efforts to fit the CPS as a unifactorial model, where all items load onto one overall purpose factor, were not as successful. The items related to Beyond-the-Self did not load well on the general factor, with loadings below 0.40, stressing on the importance of viewing these three dimensions as distinct constructs (Veazey et al., 2023). These findings underscore the need to validate the CPS in culturally diverse settings. Dimensions like Beyond-the-Self may be interpreted differently depending on the cultural context. As a result, the CPS can be considered as a valid and reliable instrument for assessing the elements of PIL, potentially enhancing the empirical study of this concept. However, to date, no validated Arabic version exists for use among Arabic-speaking adolescents.

Although the construct of PIL has been significantly explored across cultures, its expression is culturally shaped rather than universally uniform. In Arabic-speaking cultures, the experience and expression of purpose are culturally embedded and tied to familial responsibilities, communal virtues, and religious frameworks (Abdel-Khalek, 2019). Adolescents in Arab cultures are usually steered by societal norms and parental expectations. Dissimilar to the individualistic nature of western societies, the pursuits of Arab adolescents may align more with communal and religious duties (Abdel-Khalek, 2019). Family is a central axis for Arab adolescents with an emphasis in tradition as a compass for social life. These values inform youths' perceptions of purpose, often appearing in aspirations to secure education or employment as a means to support their families (Abdelmoneium et al., 2023). Hence, adapting and validating a tool such as the CPS is fundamental for ensuring that assessment of purpose accurately reflect the cultural realities of Arabic-speaking adolescents.

Given the identified cultural differences and the lack of validated tools in Arabic-speaking populations, this study aims to bridge the gap by validating the CPS in an Arabic-speaking, Lebanese adolescent sample. In the Arab world, adolescents face a range of difficulties, such as mental health challenges, unhealthy eating habits, lack of exercise, and increased exposure to violence. These difficulties are made worse by the socio-political turbulences (Obermeyer et al., 2015). In light of these obstacles accurately measuring PIL is crucial not only for evaluating teen health, but also for enacting impactful interventions. The CPS stands out as a tool in this setting. It offers a multidimensional assessment of purpose that well relates in an intrinsic manner to resilience and satisfaction with life (Bronk et al., 2018). In this context, the main objective of our study is to validate the use of the CPS. By achieving this, we hope to shed light on how cultural differences impact the way how Arabic speaking adolescents express and measure their sense of purpose. It is expected that the Arabic translation of the CPS will demonstrate good reliability and validity in a sample of Arabic speaking adolescents.

## Methods

### Participants and procedure

Data for this cross-sectional study was collected via an online link during November 2023. Potential participants were approached by the research team and asked to complete the questionnaire. Following the snowball sampling technique adopted, participants were then asked to forward the link of the survey to others they might know. Inclusion criteria for the study required participants to be Lebanese adolescents and residents aged between 14 and 18 years. The introductory paragraph included information about the study, as along with a request to the adolescent to ask for parental permission before filling the survey. After providing digital informed consent, the participant was instructed to complete the following sections in the Google Form. No personally identifiable information was gathered to ensure anonymity. Participation was on a voluntary basis. No incentives were offered for participating in the study.

### Measures

The questionnaire contained questions about age, gender, household crowding index (reflecting the socioeconomic status of the family and calculated by dividing the number of persons by the number of rooms in the house except the kitchen and bathrooms (Melki et al., 2004) as well as other scales as follows:

#### The Claremont purpose scale (CPS)

The CPS questionnaire, developed by Bronk et al. (2018) (Bronk et al., 2018) consists of 12 questions aimed at evaluating three aspects of purpose in individuals' lives. The first section, General Meaning evaluates how much a person perceives their life to be meaningful or purposeful. The second section, Goal Directedness gauges the degree to which an individual has long term goals that drive them with values-based motivation. The third section, Beyond the Self assesses how much someone seeks to influence others and the world at large. Each section includes four questions. Participants rate their responses on a 5-point Likert scale ranging from 1 = strongly disagree to 5 = strongly agree; although all responses fall within this range the wording varies for each question. The CPS provides a score and three subscale scores. The total scores range from 12 to 60 while the subscale scores range from 4 to 20. Higher scores signify increased levels of perceived purpose, general meaning, goal directedness and purpose beyond oneself. For this study, the scale was translated into Arabic following a rigorous translation-back-translation process to maintain the semantic integrity of the scale items. This involved translating the scale into Arabic following international standards to ensure semantic equivalence with the original measure (Van Widenfelt et al., 2005). Notably, the scale was translated into the modern standard Arabic (not the Lebanese dialect), a standardized literary Arabic which can be read and easily understood by Arabic speakers in all parts of the world. The standard Arabic is also the official language adopted in all 22 Arab countries of the MENA region. We employed the forward-backward translation technique. The forward translation process was performed first by a Lebanese translator who did not have any ties to our study, Then, the backward translation was done by a proficient Lebanese psychologist. The translations were carefully balanced for specificity (Azzi et al., 2023; Fekih-Romdhane et al., 2023c). A committee of experts (composed of the researchers, the two translators, one psychologist and two psychiatrists) compared the translated versions to the original one to address any ambiguities. An adaptation of the measure was then performed to suit the particular Arab context, with the specific aim to verify the ease of interpretation of the items and identify any misunderstanding regarding the wording of each item. The adaptation sought to detect and rectify misunderstandings in interpretation and wording, ensuring conceptual equivalence. During the translation and adaptation processes of the CPS, no modifications of the item wording were incorporated or deemed to be incorporated to better fit the Lebanese context, and no revision of the type/number of anchor points was made. A pilot study with 30 participants confirmed the clarity of all items. Those participants did not indicate any ambiguity, offensiveness of meaning or unclarity; therefore, no changes were deemed necessary to the CPS.

#### The brief irritability test (BITe)

The BITe is self-administered and contains five items assessing irritability over the past 15 days. Items are scored on a 6-point Likert scale from 1 (never) to 6 (always). The scale's items ask respondents to rate their recent experiences of irritability-related feelings. Greater scores indicate higher levels of irritability. The BITe demonstrates excellent internal consistency, where the Cronbach's alpha for the five items was 0.88, indicating high internal reliability (Holtzman et al., 2015). Additionally, this scale has shown good psychometric properties in its Arabic-language validated version used in our current research (Fekih-Romdhane et al., 2023a).

#### The short depression-happiness scale (SDHS)

The Short-Depression Happiness Scale is a six-item scale used to measure a person's wellbeing on the depression-happiness spectrum (Joseph et al., 2004). The scale includes three negative items like “I felt dissatisfied with my life” and three positive ones such as “I felt that life was enjoyable.” Items can be scored on a four-point Likert scale ranging from 0 (never) to 3 (often). A lower score on the scale signifies happiness and greater level of depression while a higher score indicates higher levels of happiness and absence of depression. The SDHS has demonstrated high internal consistency across multiple populations, with Cronbach's alpha values ranging from 0.80 to 0.90, indicating excellent reliability (Joseph et al., 2004). Moreover, The SDHS has shown strong convergent validity, correlating well with other established measures of wellbeing, life satisfaction, and mental health (Joseph et al., 2004). Its validity is further backed by its capability to differentiate between individuals with high levels of happiness and those experiencing depression (Joseph et al., 2004).

#### The world health organization- five wellbeing index (WHO-5)

This is is a 5-item short measure assessing self-reported levels of subjective wellbeing (Topp et al., 2015). The WHO-5 scale considers positive wellbeing to be another term for mental health, thus only contains positive phrases (e.g., *I have felt calm and relaxed*). The respondent will rate how well these phrases apply to them when thinking about the past 14 days. Items are rated on a 6-point Likert scale (0 to 5). The score ranges from 0 (absence of wellbeing) to 25 (maximal wellbeing). The WHO-5 has shown high internal consistency across multiple studies and populations. Additionally, this scale has been validated in Arabic (Fekih-Romdhane et al., 2023b).

### Statistical analysis

One-factor, three-factor, and second-order models were tested using CFA with a WLSMV (Weighted Least Squares Mean and Variance Adjusted) estimator. WLSMV is the most adequate estimator for ordinal (Likert) data. These models were assessed using the “lavaan” package for R. To perform the CFA based on 3 to 20 times the number of the scale's variables, a minimum sample of 240 participants was deemed necessary (Mundfrom et al., 2005). Calculated fit indices were the normed model chi-square (χ^2^/*df*), the Tucker-Lewis Index (TLI), the standardized root mean square residual (SRMR), the root mean square error of approximation (RMSEA), and the comparative fit index (CFI). Values ≤ 5 for χ^2^/*df*, ≤ 0.08 for RMSEA, ≤ 0.05 for SRMR and ≥ 0.90 for CFI and TLI indicate good fit of the model to the data (Hu and Bentler, 1999). Multivariate normality was not verified at first (Bollen-Stine *p* = 0.002; Critical ratio >5); supporting the use of a robust estimator (WLSMV) that does not assume multivariate normality or homoscedasticity. There were no missing responses in the dataset.

### Measurement invariance across sex

A multi-group CFA was intended to investigate sex invariance of CPS scores (Chen, 2007). Measurement invariance was explored at the metric, configural, and scalar levels (Vandenberg and Lance, 2000). As evidence of invariance, ΔCFI ≤ 0.010 and ΔRMSEA ≤ 0.015 or ΔSRMR ≤ 0.010 were accepted (Chen, 2007). Comparison between sexes was performed using the Student's *t*-test only if scalar or partial scalar invariance.

Composite reliability in the two subsamples was investigated using Cronbach's alpha and McDonald's ω, with values > 0.70 reflecting appropriate reliability (Malkewitz et al., 2023). Normality of the CPS score was verified since the skewness and kurtosis values for each item of the scale varied between −1 and +1 (Hair et al., 2017). To assess concurrent validity, Pearson test was used to correlate two scores.

## Results

**Figure 1:**
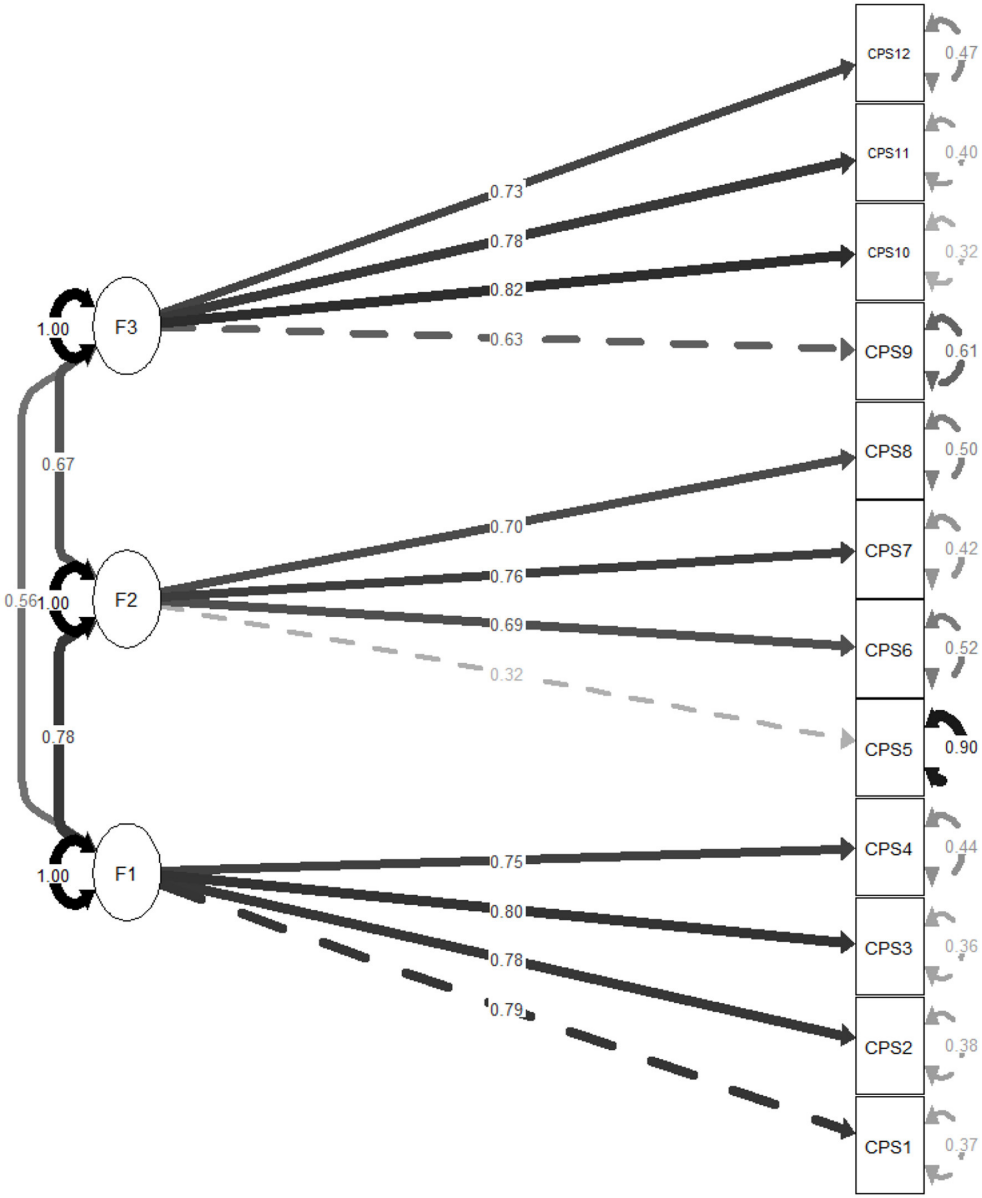
Standardized loading factors of the three-factor model of the Claremont Purpose Scale (CPS). F1 = Meaningfulness; F2 = Goal Orientation; F3 = Beyond-the-self dimension.

**Figure 2:**
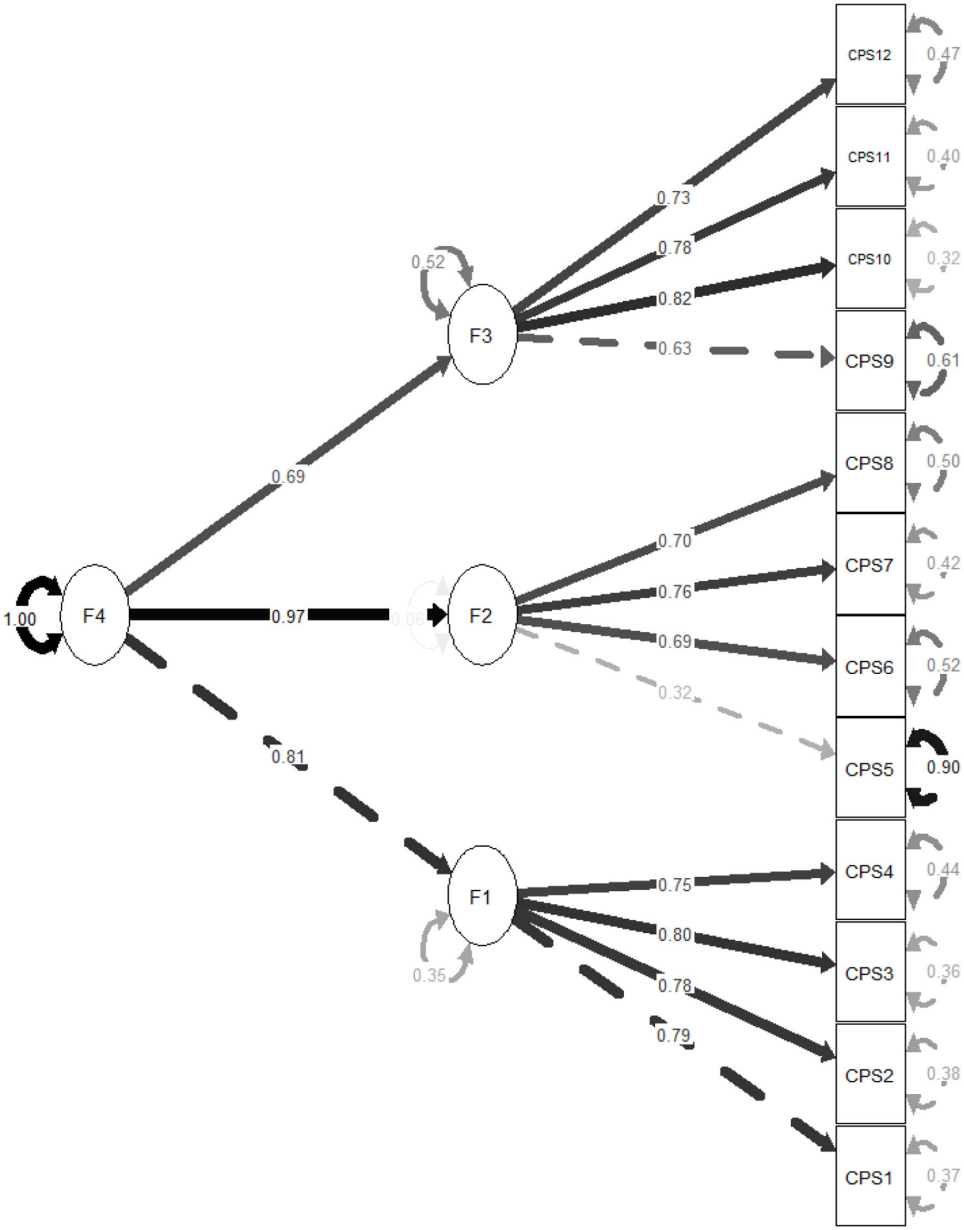
Standardized loading factors of the hierarchical (second-order) model of the Claremont purpose scale (CPS). F1 = Meaningfulness; F2 = Goal Orientation; F3 = Beyond-the-self dimension.

**Figure 3:**
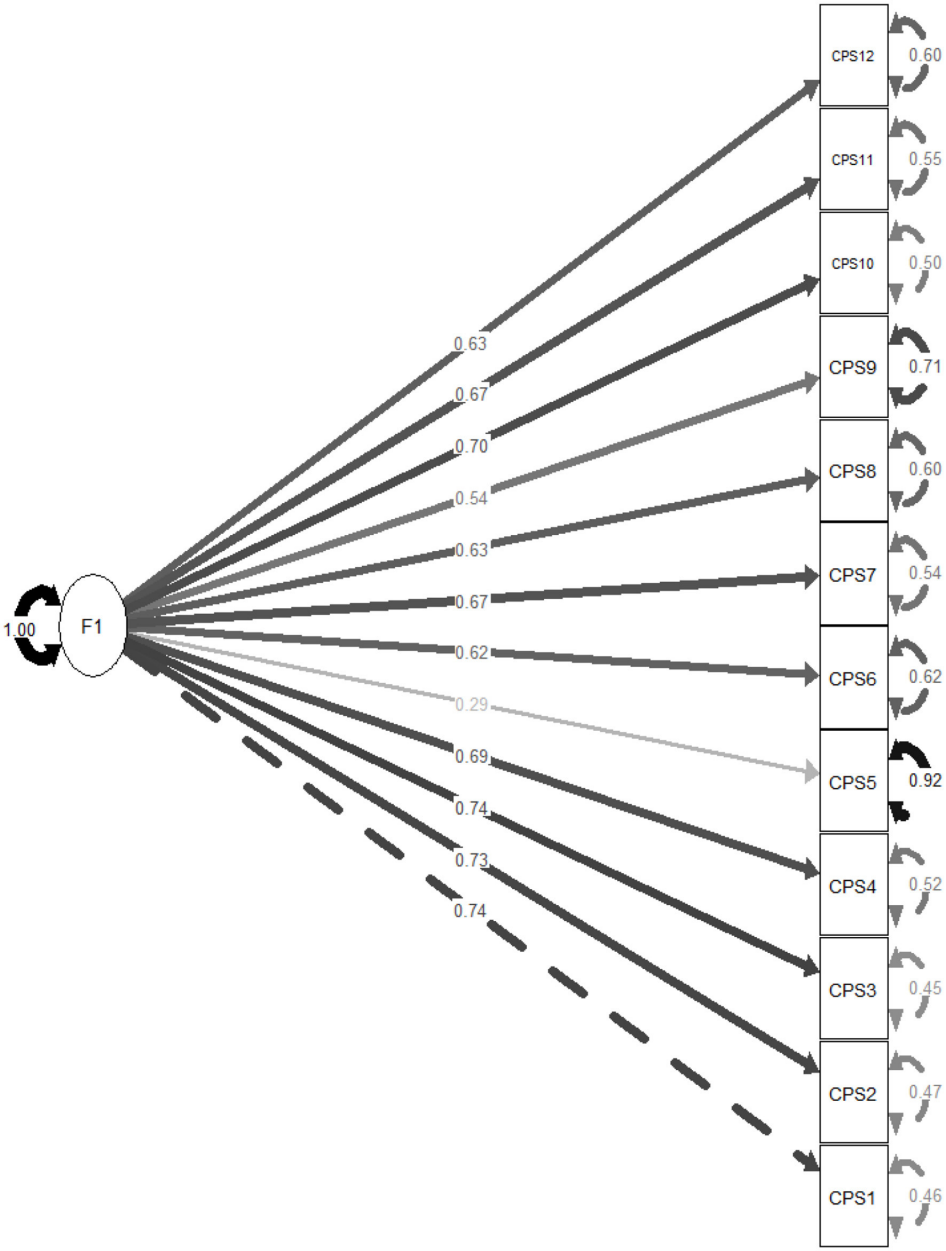
Standardized estimates of factor loadings of the one-factor model of the Claremont purpose scale (CPS).

A total of 793 adolescents (62.4% females) took part in the study. The mean age of our participants was of 16.08 ± 1.74 years. CFA showed that the fit of the three-factor model of CPS scores was adequate: scaled χ^2^/*df* = 213.86/51 = 4.19, RMSEA =.063 (90% CI 0.055, 0.072), SRMR = 0.035, CFI = 0.979, TLI = 0.972. The standardized estimates of factor loadings were all adequate (Figures 1–3). Internal reliability was excellent (ω = 0.87; α = 0.86). The second-order CFA also showed adequate fit: scaled χ^2^/*df* = 213.86/51 = 4.19, RMSEA = 0.063 (90% CI 0.055, 0.072), SRMR = 0.035, CFI = 0.979, TLI = 0.972. The one-factor model showed a reduction in model fit: scaled χ^2^/*df* = 1,389.66/54 = 25.74, RMSEA = 0.177 (90% CI 0.169, 0.185), SRMR = 0.091, CFI = 0.825, TLI = 0.787. A *post hoc* power analysis using the semPower package (Moshagen and Bader, 2024) indicated excellent power (1 – β > 0.99) to detect moderate model misfit (RMSEA = 0.05) with 51 degrees of freedom and *N* = 793.

### Sex invariance

Indices suggest that metric, configural and scalar invariance are supported across sex groups (See Table 1 for fit indices). Additionally, no significant differences were observed between female and male participants in terms of CPS scores (37.76 ± 7.92 vs. 37.20 ± 7.93, *t*_(791)_ = 0.97, *p* = 0.334), supporting invariance between sexes.

**Table 1:** Measurement Invariance across sex in the total sample.

**Model**	**Scaled CFI**	**Scaled RMSEA**	**Scaled SRMR**	**Model Comparison**	**ΔCFI**	**ΔRMSEA**	**ΔSRMR**
Configural	0.972	0.074	0.043				
Metric	0.978	0.063	0.046	Configural vs. Metric	0.006	−0.011	0.003
Scalar	0.979	0.065	0.046	Metric vs. Scalar	0.001	0.002	<0.0001

CFI, comparative fit index; SRMR, standardized root mean square residual; RMSEA, Steiger-Lind root mean square error of approximation.

### Concurrent validity

The CPS total score was significantly correlated with greater depression-happiness (*r* = 0.43; *p* < 0.001), higher wellbeing (*r* = 0.51; *p* < 0.001), and lower irritability (*r* = −0.66; *p* < 0.001).

## Discussion

The CPS serves as a comprehensive tool for evaluating PIL in adolescents by measuring goal-directedness, personal meaning, and self-transcendence, supporting identity development (Bronk et al., 2018). The study results support the validity and reliability of the Arabic version of the CPS when administered to adolescents. CFA indicates that the original three-factor structure of the CPS has good fit indices. These results are consistent with other research that highlighted the scale's good internal reliability and model fit in both Western and non-Western contexts (Bronk et al., 2018; Wang et al., 2023). For example, in the seminal validation paper, the CPS showed that all items fell onto three dimensions with excellent internal consistency (α = 0.916–0.935) among adolescents from the United States. The Chinese version also had a three-factor structure with excellent model fit and good internal consistency (Wu et al., 2024). Additionally, a study testing an Indonesian translation of the CPS found adequate fit to the data of the model based on the original version, as well as a satisfactory internal consistency of the total score and three subscales scores (Yuliawati, 2022). This reflects that the scale maintains adequate psychometric properties in the new cultural context. The latter is key for the application of the CPS in different environments.

Further, the CPS was characterized by high internal consistency, reflected by McDonald's ω (0.87) and Cronbach's α (0.86). The above high-level reliability proves that CPS is a consistent and stable measure for the assessment of PIL amidst Arabic-speaking adolescents, as parallel to other studies done in the world, which include China (α = 0.90; Wang et al., 2023) and the United States (α = 0.878; Veazey et al., 2023).

The findings on sex invariance are significant and in alignment with previous research (Bronk et al., 2018; Yuliawati, 2022; Veazey et al., 2023). These results would indicate strongly that the scale demonstrated invariance across sex at the configural, metric, and scalar levels. Invariance at the configural level reflects structural equivalence, or the fact that the same model holds for both males and females. Invariance at the metric level indicates that factor loadings are the same across sex groups. Invariance at the scalar level signifies that the intercepts are the same across male and female respondents being compared, thus indicating full score equivalence (Putnick and Bornstein, 2016). Overall, our findings confirm that the CPS measures purpose equivalently among male and female adolescents, hence becoming a strong tool in the comparison of cross-sex data in research setting. Moreover, CPS scores correlated as expected with psychological constructs such as irritability, depression, happiness, and overall wellbeing. These are consistent with previous findings that show that high scores on the CPS have been found to correspond to less depression, less irritability, and with higher happiness and wellbeing (Veazey et al., 2023). This concurrence validity further solidifies the effectiveness of CPS as a measure of positive psychological outcomes and the utility in the application of mental health assessments amongst adolescents.

## Implications

The results of this study add to the mounting body of literature supporting universality in the construct of PIL and extending its applicability to Lebanese adolescents (Bronk et al., 2018; Yuliawati, 2022; Veazey et al., 2023). Practically, the CPS will greatly help educators and clinicians assess and intervene in leading a PIL related to better mental health consequences, such as low irritability and high wellbeing (Bronk et al., 2018). It further underscores the implications of cultural sensitivity on psychological measurement. A successful adaptation and validation of the CPS in the Arabic context, therefore, also illustrates how there might be other psychological instruments that can be adapted successfully through this manner and hence increase their utility and relevance in non-Western cultures. Although items were not compulsory modified, responses to items within the Beyond-the-Self domain may have been influenced by cultural factors, such as religious and familial duties. This could demonstrate that the construct of self-transcendence may be expressed through communal responsibilities rather than global altruism, potentially offering insight for creating culturally grounded theoretical frameworks in the future.

## Limitations and future research

Despite its strength, the following study has certain limitations that need to be considered in light of future investigations. First, recruitment of participants was done utilizing online recruitment and the snowball sampling technique. This approach may overrepresent specific demographic groups (e.g., adolescents with similar socioeconomic or educational backgrounds) and limit the generalization of the findings into other populations and age groups among Arabic-speaking countries. This issue is salient and needs to be addressed in future research, as such sociodemographic variables were shown to significantly affect PIL among adolescents (Pickett et al., 2025). While we conducted a rigorous translation and back-translation process with bilingual experts and reviewed the items with a panel of professionals in psychology and education to ensure conceptual and cultural relevance, we acknowledge that cognitive interviews and differential item functioning analysis were not performed in the study. The study recommends further that the findings of this study be replicated in other diverse settings of the Arabic-speaking countries and with the out-of-school youth. Second, a self-report survey was used to gather data from participants, which may have led to some degree of response and desirability biases. Longitudinal research will need to critically assess whether the CPS is stable over time and makes valid predictions with respect to future indicators of PIL, such as its continuity in long-lasting projects, contributions to society, influence on academic/career achievements and consequences for mental health.

## Conclusion

In summary, the Arabic translation of the CPS has demonstrated reliability and validity in assessing the sense of purpose in adolescent Arabic-speakers. Therefore, it would be valuable for future researchers and practitioners working on improving the psychological functioning of youth from diverse cultural backgrounds. The scale holds considerable potential for future research to further extend its validity, thus increasing its applicability and relevance across a wider range of demographic and cultural contexts.

## Data Availability

The raw data supporting the conclusions of this article will be made available by the authors, without undue reservation.
